# High somatic mutation and neoantigen burden are correlated with decreased progression-free survival in multiple myeloma

**DOI:** 10.1038/bcj.2017.94

**Published:** 2017-09-22

**Authors:** A Miller, Y Asmann, L Cattaneo, E Braggio, J Keats, D Auclair, S Lonial, The MMRF CoMMpass Network, S J Russell, A K Stewart

**Affiliations:** 1 Department of Molecular Medicine, Mayo Clinic, Rochester, MN, USA; 2 Center for Individualized Medicine, Mayo Clinic, Rochester, MN, USA; 3 Division of Biomedical Informatics, Department of Health Sciences Research, Mayo Clinic, Jacksonville, FL, USA; 4 Division of Hematology and Oncology, Mayo Clinic, Scottsdale, AZ, USA; 5 Translational Genomics Research Institute, Phoenix, AZ, USA; 6 Multiple Myeloma Research Foundation, Norwalk CT, USA; 7 Department of Hematology and Medical Oncology, Emory University, Atlanta, GA, USA; 8 Department of Hematology, Mayo Clinic Rochester, MN, USA

## Abstract

Tumor-specific mutations can result in immunogenic neoantigens, both of which have been correlated with responsiveness to immune checkpoint inhibitors in highly mutagenic cancers. However, early results of single-agent checkpoint inhibitors in multiple myeloma (MM) have been underwhelming. Therefore, we sought to understand the relationship between mutation and neoantigen landscape of MM patients and responsiveness to therapies. Somatic mutation burden, neoantigen load, and response to therapy were determined using interim data from the MMRF CoMMpass study (NCT01454297) on 664 MM patients. In this population, the mean somatic and missense mutation loads were 405.84(*s*=608.55) and 63.90(*s*=95.88) mutations per patient, respectively. There was a positive linear relationship between mutation and neoantigen burdens (*R*^2^=0.862). The average predicted neoantigen load was 23.52(*s*=52.14) neoantigens with an average of 9.40(*s*=26.97) expressed neoantigens. Survival analysis revealed significantly shorter progression-free survival (PFS) in patients with greater than average somatic missense mutation load (*N*=163, 0.493 vs 0.726 2-year PFS, *P*=0.0023) and predicted expressed neoantigen load (*N*=214, 0.555 vs 0.729 2-year PFS, *P*=0.0028). This pattern is maintained when stratified by disease stage and cytogenetic abnormalities. Therefore, high mutation and neoantigen load are clinically relevant risk factors that negatively impact survival of MM patients under current standards of care.

## Introduction

Multiple myeloma (MM) is characterized by the clonal expansion of malignant plasma cells in the bone marrow, for which recent therapeutic advances have extended overall survival (OS), but most patients ultimately relapse.^1, 2^ There is, however, variability in response between patients and survival is known to vary with prognostic factors, including disease stage at diagnosis and cytogenetic abnormalities.^3^ Therefore, correlating prognostic factors with biological function could underpin the development of novel and targeted therapeutic classes.

One novel class of agents currently explored in clinical trials for MM is immunotherapeutic checkpoint inhibitors. Single-agent checkpoint inhibitors can reverse tumor-induced downregulation of T-cell function by blocking the engagement of checkpoint receptors like cytotoxic T-lymphocyte-associated protein 4 (CTLA-4) and programmed-death-1(PD-1) on the surface of T-cells with their cognate ligands expressed on tumor cells.^4, 5, 6^ This allows activation of the cytotoxic T-cell via engagement of the T-cell receptor (TCR) with antigenic peptides presented in the class I major histocompatibility complex (MHC) on tumor cells.

For this reason, the presence of tumor-specific antigenic peptides, or neoantigens, is thought to enhance the efficacy of cancer immunotherapy by providing endogenous tumor-specific immune targets. Neoantigens have the potential to be loaded into MHC class I molecules and presented to cytotoxic T-cells as immunogenic targets that can generate an anti-tumor immune response.^7^ Cytotoxic T-cells activated against tumor neoantigens can kill tumor cells presenting mutagenic peptides and lead to anti-tumor immunological memory that resists tumor recurrence. Somatic mutations in coding regions of the genome have the potential to generate neoantigens, thereby influencing patient response to targeted immunotherapies. A patient’s neoantigen burden can be predicted bioinformatically using algorithms to predict MHC binding affinity of mutagenic peptides translated from somatic mutations.^8, 9^ Although mutational burden in cancer is heterogeneous, tumor types are associated with a characteristic mutational load.^10^ Within cancer populations, high somatic mutation loads have been correlated with increased genomic instability, resistance to therapy, and decreased overall survival.^11, 12, 13^ Interestingly, somatic mutation load has also been correlated with neoantigen load in many cancer types, and both have been correlated with prolonged survival with checkpoint inhibitors in highly mutagenic advanced melanoma and lung cancer.^14, 15, 16^

However, correlation of survival with mutation and neoantigen burden has not been reported for cancers of lower mutational burden such as MM. Early results of single-agent checkpoint inhibitors in MM are underwhelming. Single-agent nivolumab (anti-PD-1 monoclonal antibody) had no objective responses in 27 MM patients,^17^ although some combination therapies of pembrolizumab (anti-PD-1 antibody) with lenalidomide and dexamethasone and with pomalidomide and dexamethasone have shown potential with greater than expected progression-free survival (PFS) and overall response rates respectively.^18, 19^ Given the seeming lack of single-agent immune responsiveness in the MM population, we sought to elucidate the underpinning reasons by first defining baseline mutation and neoantigen burden in patients, and assessing the association with time to progression on current therapies using the data from the Multiple Myeloma Research Foundation’s (MMRF) CoMMpass study (Relating Clinical Outcomes in MM to Personal Assessment of Genetic profiles). This observational study was designed to enroll 1000 newly diagnosed MM patients and track them from initial diagnosis through treatment for 5 years, including sequential tissue sampling and molecular profiling to identify genomic and clinical characteristics that define patient subsets and correlate with treatment response (NCT01454297).^20, 21, 22^

Herein, we report mutational load and predicted expressed neoantigen load in MM patients identified using exome sequencing and HLA-binding predictions. Elevated mutation and neoantigen load were found to correlate with significantly decreased PFS time after induction therapy, indicating the need for better treatment options for this subset of patients. This provides the basis for optimizing personalized treatment approaches for MM patients based on mutation and neoantigen load.

## Materials and methods

### The MMRF CoMMpass study and sequencing analysis

The data were obtained from the ongoing MMRF CoMMpass Trial (NCT01454297), a longitudinal study in MM relating clinical outcomes to genomic profiles of selected plasma cells (greater than 80% light chain restricted, CD138+ plasma cells) from the bone marrow of newly diagnosed patients. Patients were followed from initial diagnosis through treatment and any following response, disease regression and retreatment. Demographic information, induction and subsequent lines of therapy, and survival events were recorded for correlative study applications.

At the time of this analysis, 912 patients had been accrued with the data available for interim analysis. Of these patients, 664 patients had all the necessary data available for analyzing mutation load, neoantigen prediction, and survival analysis based on the IA8 trial release from the MMRF researcher gateway portal (https://research.themmrf.org).^20, 21, 22^ The study cohort therefore consisted of these 664 patients.

Samples were collected and processed as described on the researcher portal. Briefly, tumor and constitutional samples obtained from all patients were submitted to Translational Genomics Research Institute (TGen) in Phoenix, Arizona from the MMRF CoMMpass Biobank at Van Andel Research Insitute (VARI) in Grand Rapids, Michigan for all genetic studies.

Sequencing analysis was based on the GRCh37 reference genome and gene annotations from Ensembl version 74, base reference genome hs37d5. Whole-exome sequencing was aligned using the mem module of BWAv0.7.8 and SAMTOOLSv0.1.19. Somatic events were identified using HAPLOTYPE CALLER, SEURATv2.6, STRELKAv1.0.13, and MUTECTv1.1.4.^23, 24, 25, 26, 27, 28, 29^ RNA sequencing reads were aligned using STAR 2.3.1z and SAMTOOLSv0.1.19. Expression estimates were calculated using CUFFLINKSv2.2.1, and HT-SEQv0.6.0.^24, 30, 31, 32^ HLA genotyping was performed using the BWAkit and Polysolver algorithms on exome sequencing data.^23, 33^ The algorithms had approximately 90% concordance (data not shown) therefore only results from BWAkit were used in subsequent neoantigen predictions.

### Binding affinity and neoantigen prediction

Somatic mutation load and neoantigen load are determined separately for each patient based on mutation detection and HLA allelic prediction from patient exome sequencing (supplemental methods). For patient-specific neoantigen prediction, all somatic missense mutations in genes with protein products greater than or equal to 8 amino acids in length, excluding nonstop mutations, were used to produce peptide sequences of 8, 9 and 10 amino acids containing the mutation by tiling across the peptide sequence containing each somatic missense mutation (up to 27 possible mutant peptides per somatic missense mutation). Patient-specific MHC class I binding affinity (half maximal inhibitory concentration, IC_50_) was predicted based on a neural network machine learning approach for all possible combinations of each HLA allele with every 8-, 9-, or 10-mer peptide generated from tiling mutant and corresponding wild-type peptide sequences using netMHC(v4.0).^9^ Following accepted standards of the field, IC_50_<500 nM was considered a predicted binder. Patient-specific neoantigens were defined as any unique combination of peptide sequence: HLA-allele with mutant peptide-binding affinity IC_50_<500 nM, and corresponding wild-type peptide IC_50_>500 nM. Expressed neoantigens were considered any such neoantigens with RNAseq counts ⩾1.

### Statistical considerations

Survival analysis and Cox-proportional hazard comparison was performed using R package ‘Survival’ with log-rank test and hazard ratio statistical tests for comparison.^34, 35^ An ANOVA or Welch two sample *t*-test was used for comparisons of continuous measures between prognostic risk groups. Analyses were performed in R version 3.2.0.^36^

## Results

### Mutation and Neoantigen Load

The interim data from the MMRF CoMMpass study (NCT 01454297) were used to assess mutation burden and predicted neoantigen load in a subset of 664 MM patients representative of the accrued CoMMpass population in terms of age, sex, race, international staging system (ISS) disease staging, and cytogenetic abnormality profile (Table 1).

**Figure 1:**
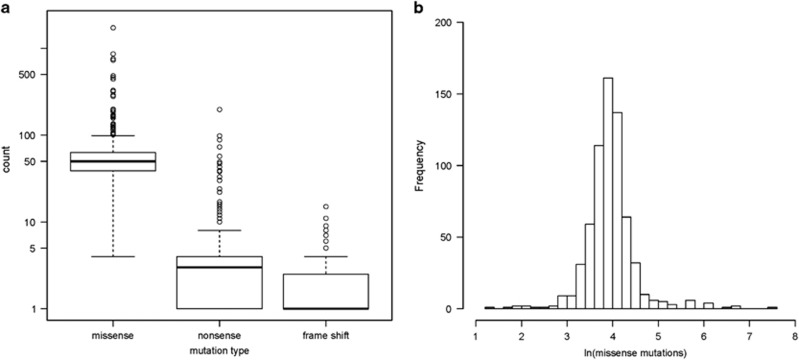
Patient-specific mutational load. (**a**) Distribution of individual patient missense, nonsense, and frameshift somatic mutation counts for the 664 patient cohort. Box represents 25–75% of patients. Bolded line marks the median mutation count. (**b**) Histogram of natural log-transformed somatic missense mutation count shows log transformation normalizes the distribution of missense mutation frequency in patients.

In order to define the predicted neoantigen burden, patient-specific mutational load and MHC class I molecules must first be determined. The human MHC class I molecules responsible for presenting neoantigens are encoded by the extremely polymorphic HLA genes at the HLA-A, HLA-B and HLA-C loci. The genomic sequencing data were used for *in silico* bioinformatic determination of the six HLA alleles carried by each patient (Supplementary Figure S1). All canonical variants reported in the CoMMpass database were used to quantify patient-specific somatic mutational load. There was an average of 405.84 (*s*=608.55) canonical somatic mutations per patient, including 38 222 unique somatic missense mutations in 12 994 unique genes across all patients. As expected, the most commonly mutated genes included immunoglobulin heavy chain and light chain genes, *NRAS, KRAS* and *BRAF*. Somatic missense mutations accounted for an average of 92% of the quantified mutations in each patient and were therefore used as a representative metric for mutation burden (Figure 1a). The distribution of log-transformed missense mutation count followed an approximately normal distribution, allowing the cohort’s mean number of missense mutations (63.90, *s*=95.88) to serve as the threshold to separate patients with low and high mutation burden (Figure 1b).

**Figure 2:**
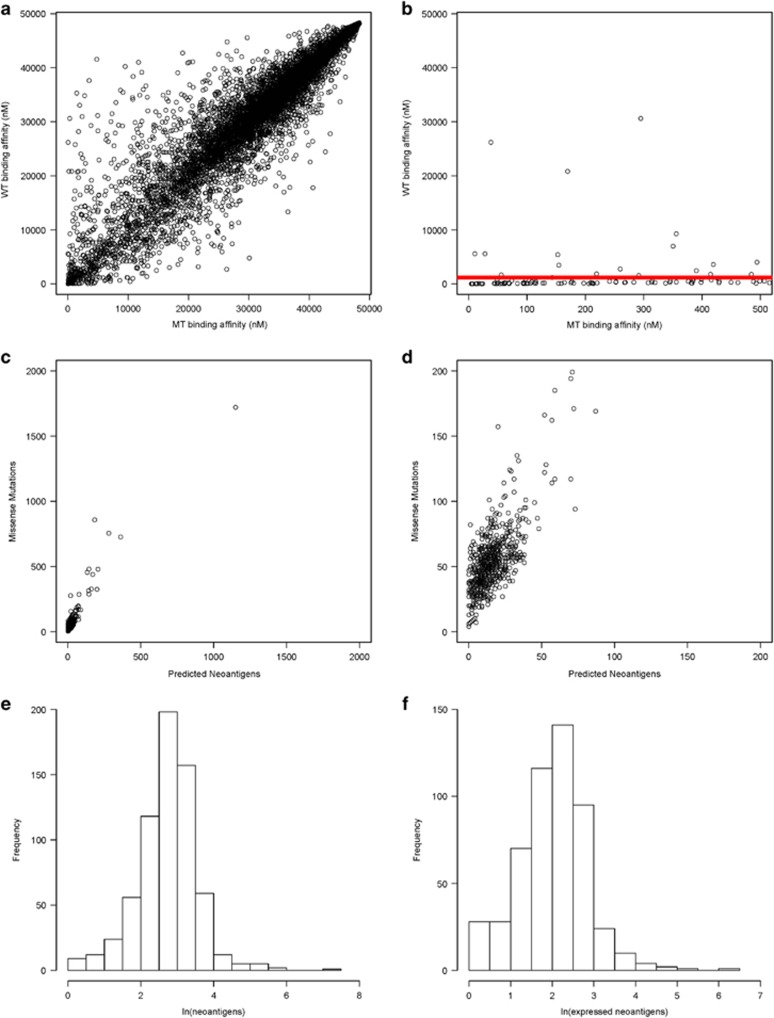
Correlation between somatic missense mutation load and predicted neoantigen load. (**a**) Example of predicted neoantigen-binding affinities relative to wild-type binding affinities in a patient with near average mutation load (missense mutation load=64). Predicted binding affinity (IC_50_) for every 8/9/10-mer peptide containing somatic missense mutation compared to the predicted binding affinity of the consensus non-mutated peptide. (**b**) Predicted neoantigen-binding affinities relative to wild-type binding affinities for the same patient after limiting the *x* axis to 500 nM. The red line indicates the accepted cutoff for potential neoantigens. Points above the red line are those with mutant peptide-binding affinity IC_50_<500 nM and wild-type peptide-binding affinity IC_50_>500 nM, and are considered potential neoantigens. (**c**) Missense mutation load vs number of potential neoantigens with predicted binding affinity IC_50_<500 nM and consensus wild-type-binding affinity IC_50_>500 nM for all multiple myeloma test cohort patients (linear regression analysis *R*^2^=0.862). (**d**) Magnified view of (**c**) using decreased x- and *y* axis limits to show missense mutation vs predicted neoantigen load in test cohort. (**e**) Histogram of natural log-transformed predicted neoantigen count (mutant peptide-binding affinity IC_50_<500 nM and wild-type peptide-binding affinity IC_50_>500 nM) shows log transformation normalizes the distribution of predicted neoantigen frequency in patients. (**f**) Histogram of natural log-transformed predicted expressed neoantigen count (mutant peptide-binding affinity IC_50_<500 nM, wild-type peptide-binding affinity IC_50_>500 nM, RNAseq counts ⩾1) shows log transformation normalizes the distribution of predicted expressed neoantigen frequency in patients.

The neoantigen load was predicted for all patients by identifying mutant peptides predicted to bind class I MHC molecules, thereby increasing the chance of antigenic presentation and T-cell activation, while the corresponding wild-type peptide has relatively weak predicted binding affinity to reduce the likelihood of immune tolerance. Comparison of mutant and corresponding wild-type peptide-binding affinity revealed the majority of binding affinities to be similar (Figure 2a). However, there is a small subset of mutant peptides with predicted binding affinity IC_50_<500 nM, widely accepted as the threshold for weak epitope binding, while the wild-type peptides have predicted binding affinity IC_50_>500 nM (Figure 2b).^37^ This filtering process, illustrated for a representative patient in Figures 2a and b, was used to identify predicted neoantigens for all patients. Henceforth, a predicted neoantigen is defined as any unique peptide:HLA combination with mutant binding affinity IC_50_<500 nM and consensus wild-type binding affinity IC_50_>500 nM. Predicted expressed neoantigens are neoantigens with evidence of expression (RNAseq count ⩾1). In this study population, the average predicted neoantigen load was 23.52 (*s*=52.14) neoantigens and 9.40 expressed neoantigens (*s*=26.97). Importantly, comparison of mutation burden and predicted neoantigen load revealed a positive linear relationship (*R*^2^=0.862, Figures 2c and d). Of the predicted neoantigens, very few are shared across the population. The most common non-immunoglobulin-based neoantigen was present in 10 patients. The approximately normal distribution of log-transformed predicted neoantigens and expressed neoantigen counts also allowed the mean neoantigen load to serve as a threshold to separate patients with low and high neoantigen burden (Figures 2e and f).

### Mutation, neoantigen load, and survival

**Figure 3:**
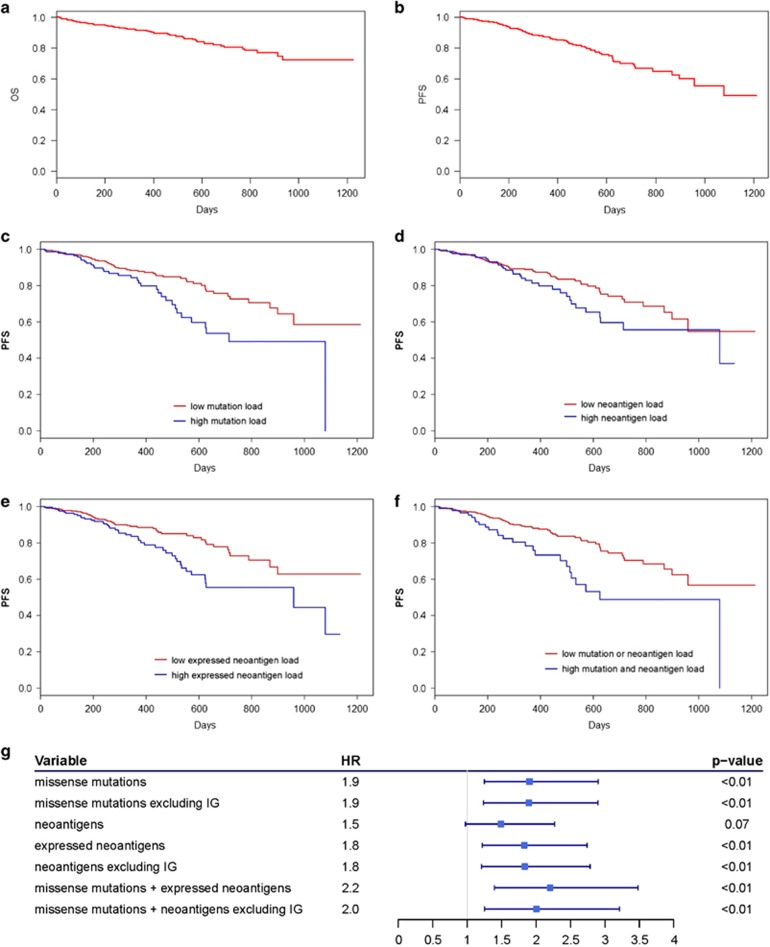
Survival in multiple myeloma patients stratified by mutation and neoantigen load. Kaplan–Meier survival curves for 664 multiple myeloma test cohort patients showing (**a**) length of overall survival (days) and (**b**) length of progression-free survival (days) after induction therapy. Univariate Kaplan–Meier survival comparison of progression-free survival (days) after induction therapy in multiple myeloma test cohort patients with (**c**) below average (low) or above average (high) somatic mutation load, (**d**) below average (low) or above average (high) neoantigens with predicted binding affinity IC_50_<500 nM and consensus wild-type binding affinity IC_50_>500 nM, (**e**) below average (low) or above average (high) expressed (RNAseq counts ≥1) neoantigens with predicted binding affinity IC_50_<500 nM and consensus wild-type binding affinity IC_50_>500 nM, and (**f**) both high somatic mutation load and high expressed neoantigen load vs those with either low somatic mutation load, low expressed neoantigen load, or both. (**g**)Cox-proportional hazard rate and corresponding forest plot associated with progression-free survival of multiple myeloma test cohort patients based on mutation burden and neoantigen burden thresholds as Cox analysis variables.

To determine the influence of mutation and neoantigen load on MM survival, it was necessary to first understand the study cohort survival. The 2-year OS rate and PFS rate for the study cohort was 0.806 and 0.667, respectively (Figures 3a and b). Due to the small number of OS events, PFS was subsequently used to evaluate impact of mutation and neoantigen load on MM survival. Univariate survival analysis revealed patients with above average (high) somatic missense mutations (*N*=163) had significantly shorter PFS (log-rank test, *P*=0.002, Figure 3c). than those with below average (low) somatic missense mutations, and a lower 2-year survival rate relative to those with low mutation load and the population as a whole (0.493 vs 0.726 and 0.667, respectively).

Similarly, patients with above average (high) predicted neoantigens (*N*=187) had shorter PFS than those with low neoantigen load (0.557 vs 0.709 PFS at 2 years, log-rank test, *P*=0.064, Figure 3d). When neoantigens are limited to those with evidence of expression, patients with high expressed neoantigen load (*N*=214) had significantly shorter PFS than those with low expressed neoantigen load (log-rank test, *P*=0.003, Figure 3e), and a lower 2-year survival rate relative to those with low expressed neoantigen loads and the population as a whole (0.555 vs 0.729 and 0.667, respectively). This relationship is maintained in individuals with both high mutation and expressed neoantigen load (*N*=98); these patients had significantly shorter PFS than patients without both high mutation and expressed neoantigen load (0.488 vs 0.703 PFS at 2yrs, log-rank test, *P*=0.001, Figure 3f).

Survival analysis was also performed excluding mutations and neoantigens arising from immunoglobulin genes since immunoglobulin genes are prone to high levels of mutagenesis, resulting in differential processing for T-cell presentation and immune tolerance.^38, 39^ Accordingly, while mutations and predicted neoantigens arising solely from immunoglobulin genes did not impact survival, patients with high mutation and neoantigen loads excluding immunoglobulin genes had significantly shorter PFS and lower 2-year survival rates than patients with low mutation or neoantigen loads excluding immunoglobulin genes (log-rank test *P*=0.003, 0.004, respectively, Supplementary Figure S2, Supplementary Table S1).

The Cox-proportional hazard model was used to determine hazard rates associated with mutation-related covariates and confirmed high mutation and expressed neoantigen load significantly increase the risk of disease progression (Figure 3g). Furthermore, the highest mutation and neoantigen burden is associated with the greatest risk of disease progression; comparison of hazard rates across incremental increases in mutation and expressed neoantigen burden revealed progressively increasing relative risk (Supplementary Figure S3). Survival analysis of patients separated by mutation and neoantigen load quartiles confirmed the shortest PFS occurs in patients with mutation and neoantigen burden in the uppermost quartile (Supplementary Figure S3).

**Figure 4:**
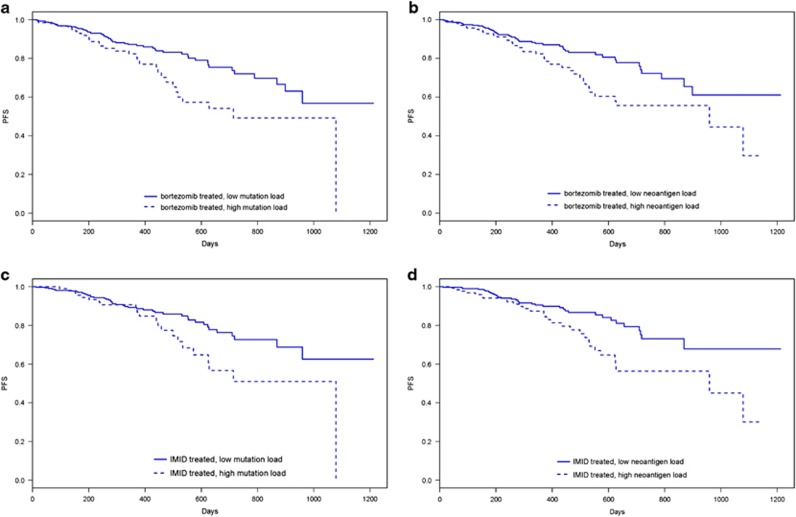
Survival in bortezomib-treated and IMID-treated multiple myeloma patients stratified by mutation and neoantigen load. Kaplan–Meier survival curves for patients from test cohort receiving bortezomib as part of first-line therapy showing length of progression-free survival (days) after induction therapy in patients with (**a**) below average (low) or above average (high) somatic mutation load, (**b**) below average (low) or above average (high) expressed (RNAseq counts ⩾1) neoantigens with predicted binding affinity IC_50_<500 nM and consensus wild-type-binding affinity IC_50_>500 nM. Kaplan–Meier survival curves for patients from test cohort receiving IMIDs (lanalidomide, pomalidomide, thalidomide) as part of first-line therapy showing length of progression-free survival (days) after induction therapy in patients with (**a**) below average (low) or above average (high) somatic mutation load, (**b**) below average (low) or above average (high) expressed (RNAseq counts ⩾1) neoantigens with predicted binding affinity IC_50_<500 nM and consensus wild-type-binding affinity IC_50_>500 nM.

To reduce possible influence of treatment type, survival was also analyzed for population subsets comprising only patients who received bortezomib and only patients who received immunomodulatory drugs (IMIDS; lenalidomide, pomalidomide, or thalidomide) as part of their induction therapy. Bortezomib-treated patients with high mutation or neoantigen load maintained a significantly shorter PFS than those with low mutation or neoantigen load (log-rank test, *P*=0.004, *P*=0.008, Figures 4a and b). Similarly, IMID-treated patients with high mutation or neoantigen load maintained a significantly shorter PFS than those with low mutation or neoantigen load (log-rank test, *P*=0.028, *P*=0.006, Figures 4c and d).

### Mutation, neoantigen load, and multivariate survival analysis

**Figure 5:**
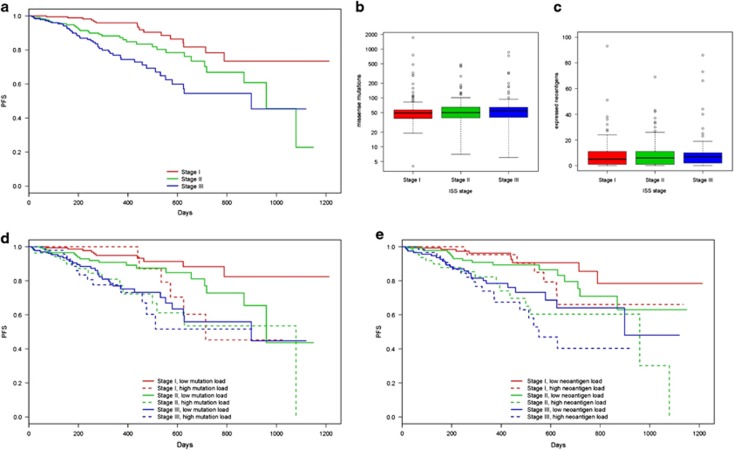
Relationship between somatic missense mutation burden, predicted neoantigen load, ISS stage and survival. (**a**) Kaplan–Meier survival curves showing length of progression-free survival (days) after induction therapy in multiple myeloma test cohort patients separated by ISS disease stage. Boxplots showing distribution of (**b**) missense mutation load and (**c**) predicted expressed neoantigen load in patients with ISS stage I, stage II, or stage III disease. (**d**) Kaplan–Meier survival curves showing length of progression-free survival (days) after induction therapy in multiple myeloma test cohort patients separated by ISS disease stage and below average (low) or above average (high) somatic missense mutation burden. (**e**) Kaplan–Meier survival curves showing length of progression-free survival (days) after induction therapy in multiple myeloma test cohort patients separated by ISS disease stage and below average (low) or above average (high) expressed neoantigen load.

We also aimed to determine the correlation between known MM prognostic risk factors and the influence of mutation and neoantigen load on survival. Disease stage, using ISS staging based on beta 2-microglobulin and albumin levels in the blood has been shown to be a reproducible predictor of MM survival.^40^ Therefore, ISS disease stage was used to determine any correlation between disease stage, mutation burden, and PFS. The correlation between advanced disease stage and poorer prognosis was evident in this cohort. Patients with later stage disease had significantly shorter PFS (log-rank test, *P*<0.001, Figure 5a) and decreased 2-year survival rates (Supplementary Table S2) compared to those with earlier stage disease. Mutation and neoantigen load does not significantly differ between patients stratified by disease stage (ANOVA *P*=0.743, *P*=0.544, respectively, Figures 5b and c), showing that mutation accumulation is not correlated with disease staging in this data set.

**Figure 6:**
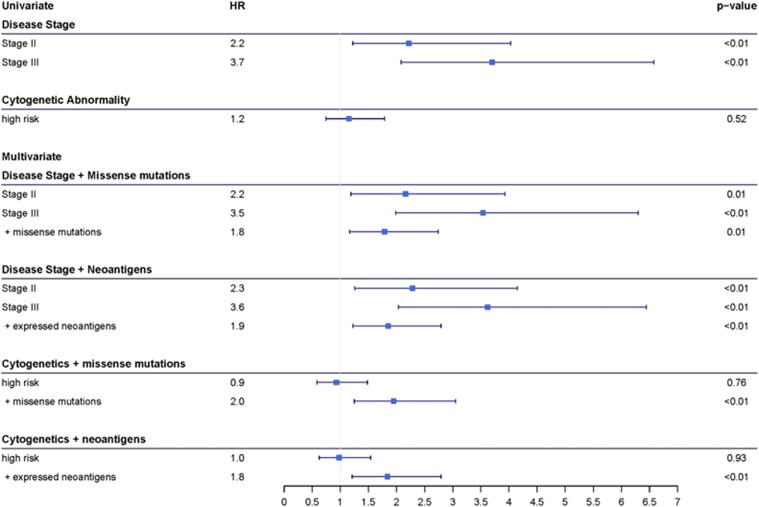
Cox-proportional hazard rate and corresponding forest plot associated with progression-free survival of multiple myeloma test cohort patients based on univariate and multivariate comparisons of disease stage, cytogenetic abnormality, and mutation and neoantigen burden as Cox analysis variables.

When survival analysis was performed on patients stratified by disease stage and mutational burden, patients with high mutation or neoantigen loads had significantly shorter PFS than those with low mutation or neoantigen loads, regardless of disease stage (log-rank test, *P*<0.001, *P*<0.001, respectively, Figures 5d and e, respectively). Patients with high mutation or neoantigen load had lower 2-year PFS rates than those with low mutation or neoantigen load in each disease stage (Supplementary Table S2). Additionally, the survival rates in patients stratified by disease stage alone are decreased when high mutation or neoantigen load is considered, while the survival rate is increased when low mutation or neoantigen load is considered (Supplementary Table S2). Cox-proportional hazard modeling revealed disease stage is significantly associated with increased risk of disease progression. Knowledge of missense mutation and neoantigen load adds significant benefit to predicting risk (ANOVA, *P*=0.010, 0.004, respectively), increasing risk of disease progression an additional 1.786- and 1.850-fold, respectively beyond the risk due to disease stage alone (Figure 6).

Analysis of additional known MM prognostic factors, including chromosome 1 amplification, elevated serum lactate dehydrogenase (LDH), and age revealed the same pattern. The correlation between each prognostic factor and decreased survival was evidenced in this study. While there was no significant difference in mutation or neoantigen load of patients with or without each prognostic factor, patients with high mutation or neoantigen loads had significantly shorter PFS and decreased 2-year survival rates compared to those with low mutation or neoantigen loads within each prognostic group (Supplementary Figures S4–S6, Supplementary Table S4–S7). Cox-proportional hazard modeling revealed all prognostic factors were significantly associated with increased risk of disease progression. The added knowledge of missense mutation and neoantigen load is significantly beneficial, increasing predicted risk of disease progression at least 1.7-fold beyond that predicted by known prognostic factors alone (Supplementary Table S8).

**Figure 7:**
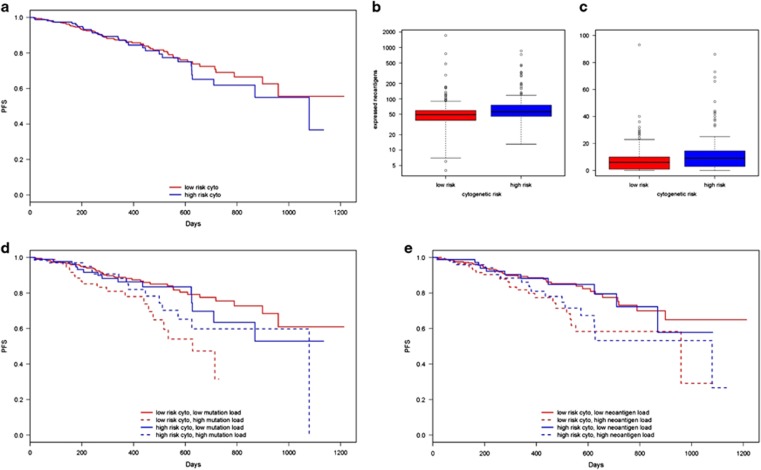
Relationship between somatic missense mutation burden, predicted neoantigen load, cytogenetic MM subtype, and survival. (**a**) Kaplan–Meier survival curves showing length of progression-free survival (days) after induction therapy in multiple myeloma test cohort patients with high-risk (t(4:14), t(14:16), t(14:20) translocations, or deletion 17p) or low-risk cytogenetic abnormalities. Boxplots showing distribution of (**b**) missense mutation load (*P*=0.005) and (**c**) predicted expressed neoantigen load in patients with high-risk or low-risk cytogenetic abnormalities (*P*=0.018). (**d**) Kaplan–Meier survival curves showing length of progression-free survival (days) after induction therapy in multiple myeloma test cohort patients with low-risk or high-risk cytogenetic abnormalities and below average (low) or above average (high) somatic missense mutation burden. (**e**) Kaplan–Meier survival curves showing length of progression-free survival (days) after induction therapy in multiple myeloma test cohort patients low-risk or high-risk cytogenetic abnormalities with below average (low) or above average (high) expressed neoantigen load.

Cytogenetic abnormalities are also associated with prognostic value. Translocations t(4;14), t(14;16) and t(14;20), and deletion 17p (*TP53*) are associated high risk of MM progression and were therefore used to separate patients into high-risk (those with one or more of any high-risk cytogenetic abnormality) and low-risk (those without any high-risk cytogenetic abnormality) groups.^41, 42^ In the current cohort, there was no significant correlation between high-risk cytogenetic abnormalities and PFS (log-rank test, *P*=0.522, Figure 7a), and patients with high-risk cytogenetics had slightly higher mutation and neoantigen load than low-risk individuals (Welch two sample *t*-test *P*=0.005, *P*=0.018, respectively, Figures 7b and c). However, consistent with other prognostic factors, when patients were stratified by cytogenetic risk and mutational burden, patients with high mutation or neoantigen loads had significantly shorter PFS (log-rank test *P*=0.005, *P*=0.030, respectively, Figures 7d and e) and lower 2-year survival rates than those with low mutation or neoantigen load in each cytogenetic risk group (Supplementary Table S3). Cox-proportional hazard modeling confirmed that mutation and neoantigen load were more beneficial in predicting risk of MM progression than cytogenetic abnormality alone (ANOVA *P*=0.005, 0.005, respectively, Figure 6).

## Discussion

Developing curative treatments for MM will require the ability to characterize and understand the implications of prognostic risk factors on patient response to therapy. Here we characterized mutation and neoantigen load in 664 MM patient cohort. We have shown that mutation load and neoantigen load are positively correlated to each other, and above average mutation or neoantigen load is a significant prognostic factor associated with increased risk of disease progression. This finding will be useful in developing targeted and individualized treatment plans that harness the immunological potential of patient mutation and neoantigen load.

Although mutation and neoantigen load have previously been correlated with response to targeted immunotherapies in highly mutated solid tumors, similar correlations have not previously been reported for cancers with characteristically lower mutation frequency, including MM.^14, 15, 16^ The lower mutational burden associated with MM relative to other cancer types was confirmed here. The average number of missense mutations in our study population, 63.9, was comparable to other MM populations.^10^ Mutational load in this population correlated positively with predicted neoantigen load, which averaged 23.52 neoantigens per patient. This study is the first to confirm the correlation of missense mutations with predicted neoantigens observed in other cancer types in the MM population, even with the more stringent neoantigen identification used here. Identification of predicted neoantigens here considered both mutant peptide and consensus wild-type-binding affinity and evidence of transcript expression to increase biological relevance in terms inducing an immune response and avoiding immune tolerance.^43, 44^ This filtering may eliminate functional neoepitopes of lower binding affinity or those where wild-type peptides are capable of binding but recognized differently by TCRs. As the field of T-cell immune tolerance advances, this definition of neoantigens may be modified to better predict tumor-specific immunological peptides.

A potential neoantigen was also defined as any unique mutant peptide:HLA allele combination. HLA determination using bioinformatic algorithms continue to be limited by the extreme polymorphic nature of the HLA loci; however, bioinformatic capabilities have improved with new prediction algorithms reporting overall accuracy near 97%.^33^ Further, binding affinity prediction algorithms do not yet support all alleles due to lack of sufficient training data.^8, 9, 45^ The neoantigens predicted here are only those bound to HLA types supported by netMHC4.0. The combination of these filters provides an accurate and thorough depiction of predicted expressed neoantigens achievable with current bioinformatic technology. While we chose to restrict our current neoantigen prediction to missense mutations with evidence of expression, as missense mutations accounted for the majority of somatic mutations identified, future work can be extended to include expressed neoantigens that have the potential to arise from other types of mutations such as frame shifts or insertions and deletions. Future experimental validation of predicted neoantigen ability to elicit immune responses is also warranted in order to apply this prognostic factor to immunotherapy development.

With recent advances in MM treatment options, the 2-year OS rate in our study population was high. For this reason, PFS after induction therapy was used as an indicator of survival outcome. Survival analysis revealed mutation and expressed neoantigen load are both significantly correlated with time to disease progression on induction therapy regardless of other prognostic risk factors. Multivariate analysis showed knowledge of disease stage, chromosome 1 amplification status, serum LDH levels, age, and mutation and neoantigen burden all significantly contribute to risk of disease progression. Knowing mutational load adds beneficial information beyond that gained by knowing other prognostic factors alone, increasing risk by at least 1.7-fold. While this cohort revealed no significant difference in survival between patients with high- and low-risk cytogenetic abnormalities, which we attribute to the limited sample of patients with the cytogenetic data and short follow-up time, multivariate analysis revealed the addition of mutation and neoantigen burden allowed differences in risk of disease progression to be predicted. Therefore, mutation and neoantigen load add significant benefit to prediction of disease risk beyond that of standard MM prognostic risk factors. It is possible that mutation and neoantigen load may be used in combination with other prognostic factors to improve predictive power such as the combination of prognostic factors in the new revised-ISS staging that combines disease stage and cytogenetic abnormalities with greater prognostic power for MM patients.^46^

We hypothesize that standard MM treatment options do not effectively generate an immune response which leverages the immunotherapeutic potential of mutation and neoantigen burden. For instance, IMIDs used for MM treatment work through anti-proliferative and anti-inflammatory but have not shown evidence of direct T-cell stimulation *in vitro*.^47^ Bortezomib is known to reduce expression of MHC class I molecules, thereby reducing the presentation of neoantigens.^48^ Indeed, analysis showed patients with above average mutation and neoantigen load treated with bortezomib or IMIDs still had significantly shorter PFS than those with below average mutational loads. We hypothesize the lack of response in MM patients to single-agent immune checkpoint blockade in early trials may be the result of inadequate patient selection and suggest high neoantigen tumors could be enriched in future studies. The association of neoantigen load with decreased survival on standard induction therapy does not preclude the role of genomic instability, DNA repair, MM clonal heterogeneity, or reduced MHC expression could have on clinical outcome and the function of neoantigens. Previous work with next-generation sequencing of MM has identified patterns in somatic mutations and differential patterns in clonal evolution that contribute to the progression of malignant MM.^49, 50, 51^ Therefore, future work will be necessary to investigate relationships between predicted neoantigens, functional immunogenicity of the neoantigens, and genomic instability on MM outcome. Nonetheless, these results identify mutation and neoantigen load as therapeutically targetable prognostic factors previously unrecognized in MM.

While mutation and neoantigen load have been correlated with prolonged survival on targeted immunotherapies in highly mutated cancers, we demonstrate a negative correlation between mutation, neoantigen load and PFS after induction therapy in MM. This identifies high mutation and neoantigen load as a clinically relevant risk factor that negatively impacts survival of myeloma patients under current standard of care. We hypothesize that current therapies are less successful for these patients because they fail to recruit T-cell immunity necessary to exploit tumor neoantigens fundamental to immune-mediated killing of cancer cells. Not only does this support the use of mutational load and neoantigen load as another MM prognostic factor, it identifies a potential unique MM subpopulation that may be targetable with immunotherapies designed to stimulate T-cell response and should be investigated in the future. This work presents an encouraging basis for pursuing targeted and personalized therapies optimized for MM patient-specific risk factors.

## Figures and Tables

**Table 1:** Demographics of complete and test cohort from interim MMRF CoMMpass population

*Demographics*	*MMRF (IA8)*	*Test Cohort*
Number of Patients	912	664
*Sex*		
Male	555, 60.86%	408, 61.45%
Female	357, 39.14%	256, 38.55%
		
*Age*		
Average	64.08	63.95
Range	27,93	27,93
		
*Ethnicity*		
Caucasian	693, 75.99%	506, 76.20%,
African American	156, 17.11%	110, 16.57%
Asian	18, 1.97%	13, 1.96%
		
*ISS stage*		
Stage I	32.3%	32.1%
Stage II	34.3%	34.2%
Stage III	29.5%	29.8%
No stage/unreported	3.8%	3.9%
		
*Cytogenetic abnormality (exome)*		
MAFB, MAF, WHSC1	13.38%	17.02%
Del17 or p53	7.89%	10.69%
None/unreported	23.68% (exome FISH)	3.92% (exome FISH)
	26.32% (long insert)	8.58% (long insert)

Abbreviation: ISS, international staging system.
