# Supplementary material for: High somatic mutation and neoantigen burden are correlated with decreased progression-free survival in multiple myeloma
Source: Blood Cancer J. 2017 Sep 22;7(9):e612–. doi: 10.1038/bcj.2017.94 (PMC5709757; doi:10.1038/bcj.2017.94)
Supplement: Supplementary Information [file bcj201794x1.docx]

**Supplemental Methods**

***Neoantigen Prediction***

All genomic data used in the prediction of neoantigens was generated and analyzed by Translational Genomics Research Institute (Tgen) for the Multiple Myeloma Research Foundation (MMRF) as described in the CoMMpass_SequencingApproach_1A10.pdf document on the MMRF research portal.

Neoantigen prediction was performed independently for each patient using whole exome sequencing data to complete *in silico* HLA allelic determination and somatic mutation calling. HLA alleles were predicted using BWAkit algorithms with independent confirmation of allele typing using Polysolver(data not shown). Patient-specific HLA alleles were compiled into a single database with unique patient identifiers for future binding affinity prediction to patient-specific peptide sequences.

Somatic mutation calling was performed by Tgen for MMRF using three different somatic variant callers SEURAT v2.6, STRELKA v1.0.13, and MUTECT v1.1.4., and accessed from MMRF database canonical variant files within somatic event files. Mutations were annotated and missense mutations were used for neoantigen prediction.

All somatic missense mutations in genes with protein products greater than or equal to 8 amino acids in length, excluding nonstop mutations, were used to produce potential neoantigen peptide sequences. First, wildtype peptide sequences were collected using BiomaRt package in R. Then, peptide sequences ≤21 amino acids in length were generated using pVACseq package ‘Generate Variant Sequences’ using patient variant information as input (chromosome name, start position, stop position, reference allele, alternative allele, gene name, ensemble transcript name, amino acid change, ensemble gene id, full wildtype amino acid sequence). Wild type and mutant peptide sequences were compiled into a single database containing information on wild type/mutant peptide label, gene ID, variant information, and ≤21mer amino acid sequence.

Patient-specific MHC class I binding affinity (half maximal inhibitory concentration, IC_50_) was predicted based on a neural network machine learning approach for all possible combinations of each HLA allele with every 8-, 9-, or 10-mer peptide generated from tiling mutant and corresponding wildtype peptide sequences using netMHC(v4.0) .[^9^](#_ENREF_9) Peptide tiling is performed by netMHC to identify all possible peptide sequences of 8, 9, and 10 amino acids containing the mutation by tiling a reading frame of corresponding length across the peptide sequence containing each somatic missense mutation (at least 8 amino acids in length and up to 21 amino acids in length to incorporate all potential peptides; up to 27 possible mutant peptides per somatic missense mutation). The corresponding wild-type genomic sequence was also used to generate wildtype consensus peptide sequences for later comparison. Binding affinity is predicted for each possible peptide:HLA allele combination. Binding affinity predictions for corresponding mutant peptide:HLA allele and wildtype peptide:HLA allele were compiled into a database for functional filtering.

All potential neoantigens were filtered for biological relevance as determined by binding affinity relative to consensus wildtype peptides to avoid immune tolerance, and evidence of expression in RNAsequencing to assure the probability of immunological presentation. Following accepted standards of the field, IC_50_<500nM was considered a predicted binder. Immune tolerance was considered possible when the wildtype consensus sequence had predicted binding affinity IC_50_≤ 500nM to the same MHC class I molecule. Therefore, patient-specific neoantigens were defined as any unique combination of peptide sequence: HLA-allele with mutant peptide binding affinity IC_50_<500nM, and corresponding wildtype peptide IC_50_>500nM. Expressed neoantigens were considered any such neoantigens with RNAseq counts ≥ 1from MMRF expression estimates.
