# Supplementary material for: High somatic mutation and neoantigen burden are correlated with decreased progression-free survival in multiple myeloma
Source: Blood Cancer J. 2017 Sep 22;7(9):e612–. doi: 10.1038/bcj.2017.94 (PMC5709757; doi:10.1038/bcj.2017.94)

Figure S1

A

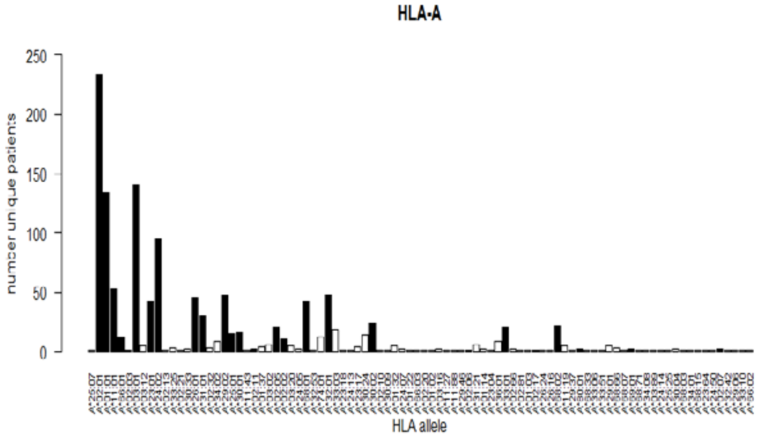

B

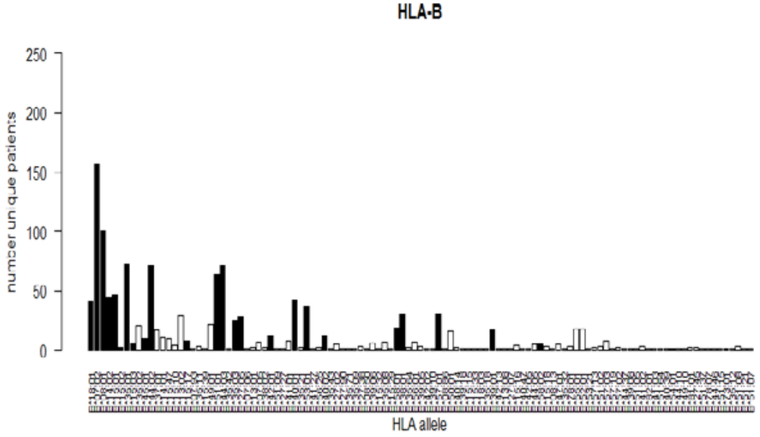

C

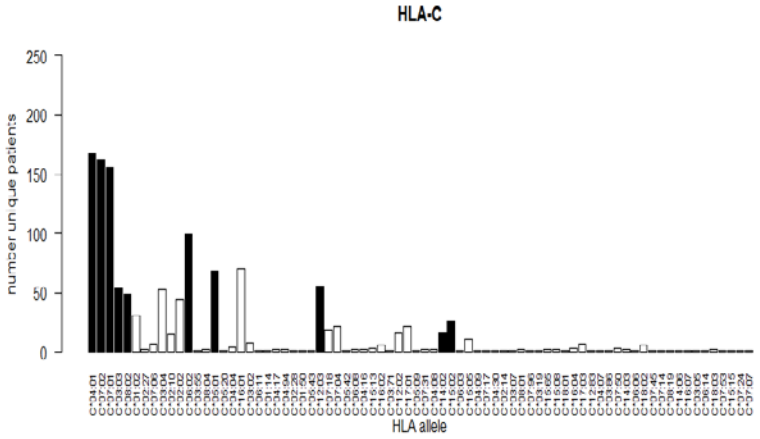

Figure S2

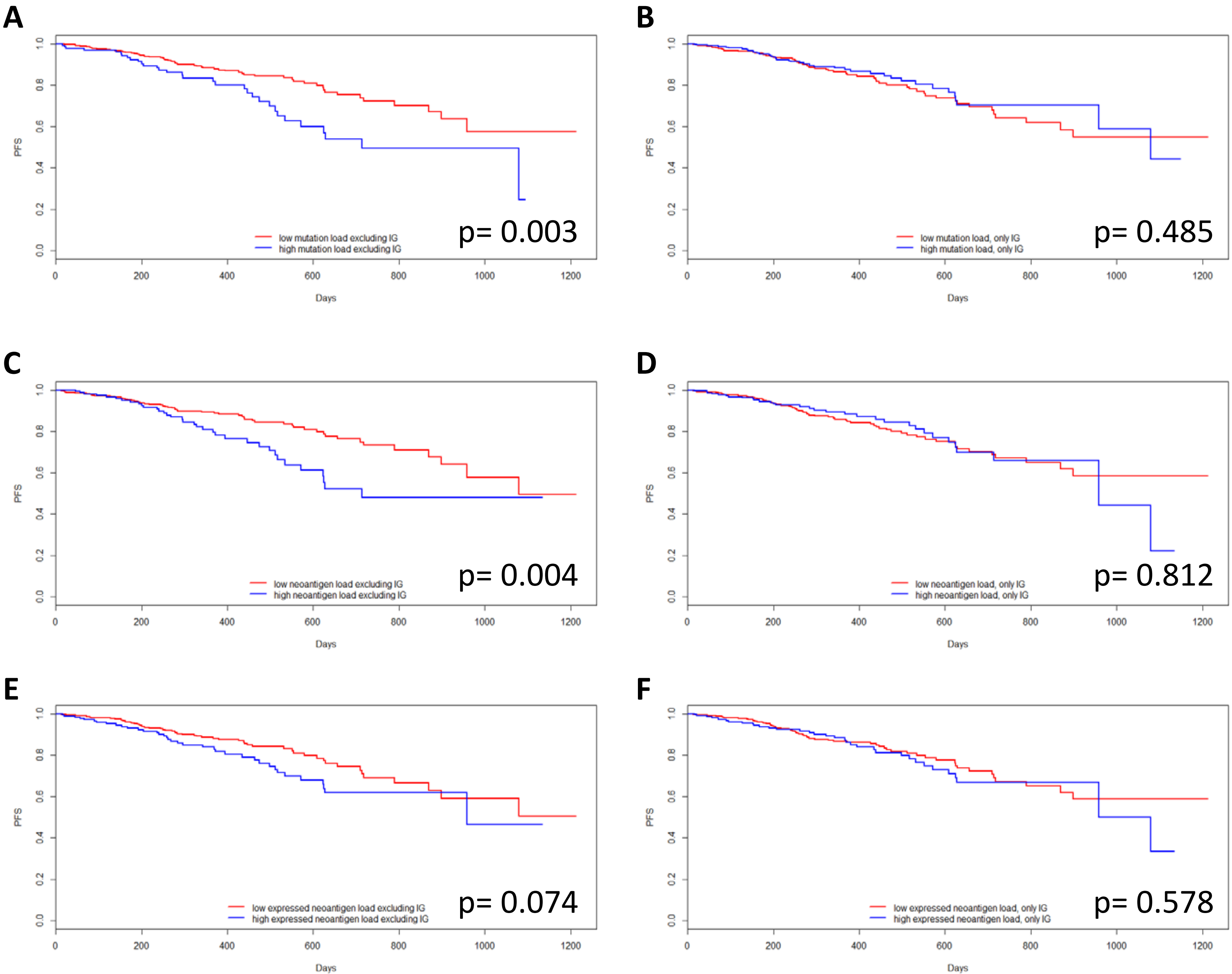
A
low mutation load excluding IG
high mutation load excluding IG
p= 0.003
B
low mutation load, only IG
high mutation load, only IG
p= 0.485
C
low neoantigen load excluding IG
high neoantigen load excluding IG
p= 0.004
D
low neoantigen load, only IG
high neoantigen load, only IG
p= 0.812
E
low expressed neoantigen load excluding IG
high expressed neoantigen load excluding IG
p= 0.074
F
low expressed neoantigen load, only IG
high expressed neoantigen load, only IG
p= 0.578
PFS
Days
0.0
0.2
0.4
0.6
0.8
1.0
0
200
400
600
800
1000
1200

Figure S3

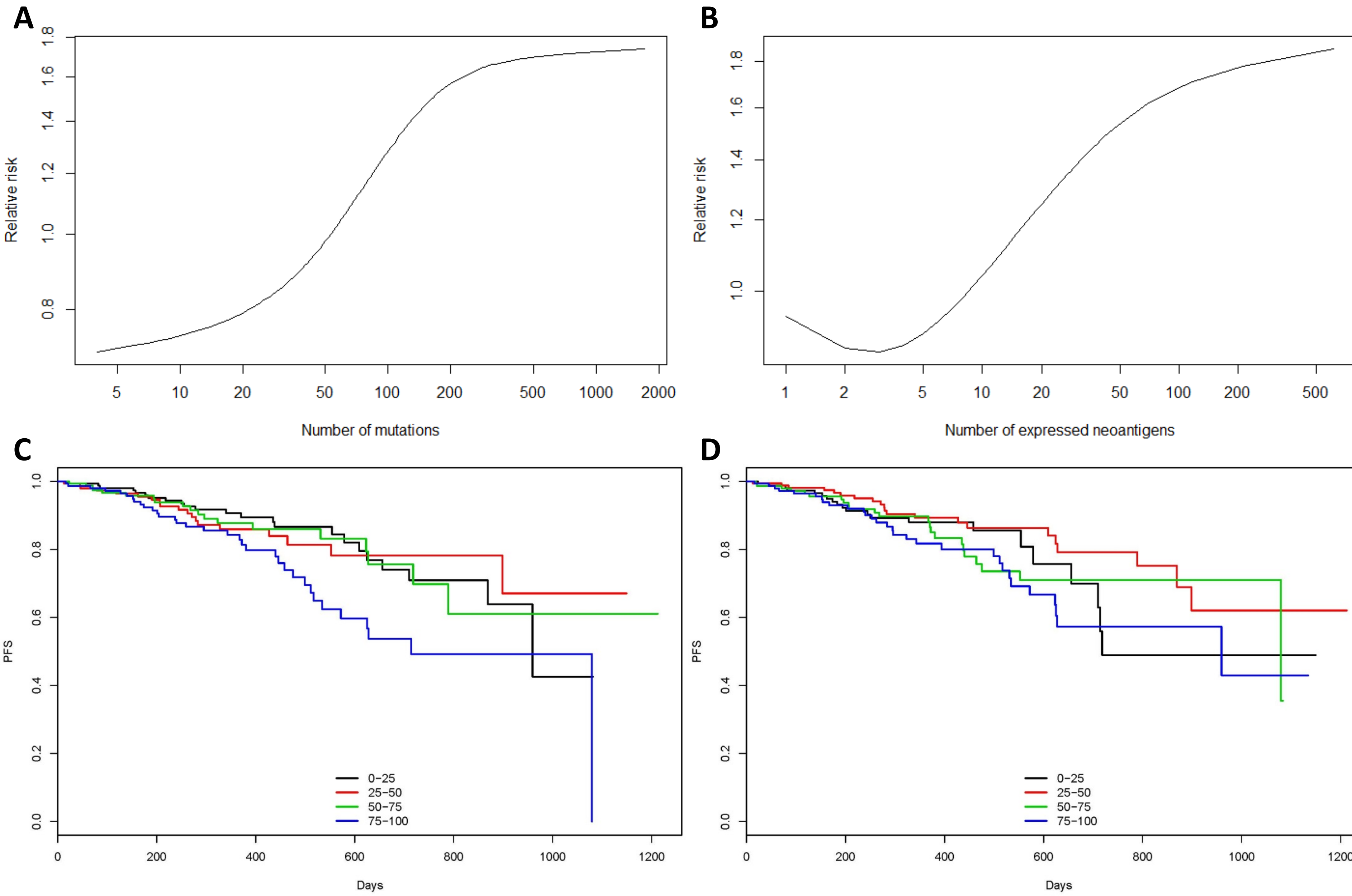
A
B
C
D
Relative risk
Number of mutations
Number of expressed neoantigens
PFS
Days
0–25
25–50
50–75
75–100

Figure S4

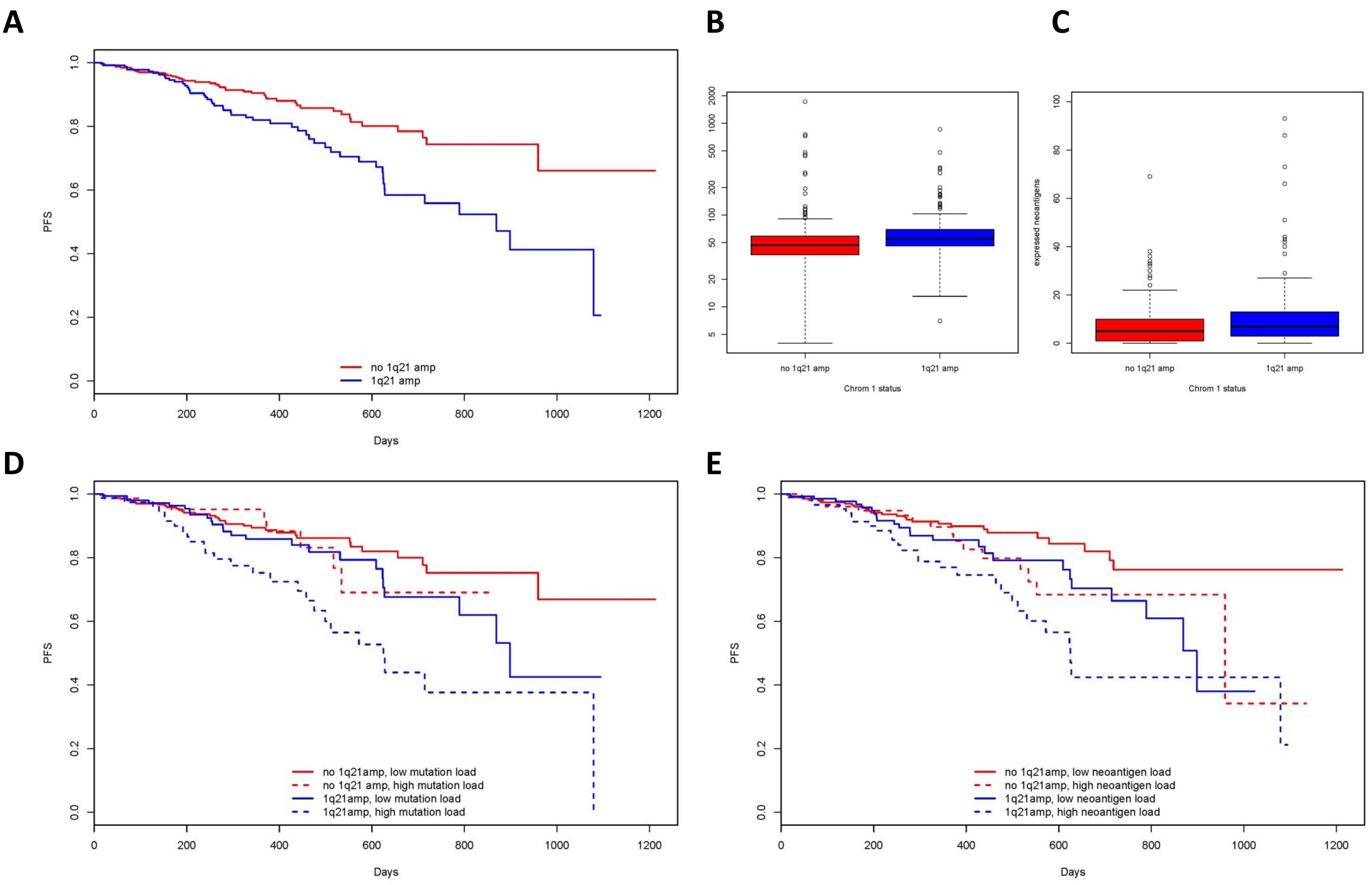

A
PFS
Days
no 1q21 amp
1q21 amp
B
no 1q21 amp
1q21 amp
Chrom 1 status
C
expressed neoantigens
no 1q21 amp
1q21 amp
Chrom 1 status
D
PFS
Days
no 1q21amp, low mutation load
no 1q21 amp, high mutation load
1q21amp, low mutation load
1q21amp, high mutation load
E
PFS
Days
no 1q21amp, low neoantigen load
no 1q21amp, high neoantigen load
1q21amp, low neoantigen load
1q21amp, high neoantigen load

Figure S5

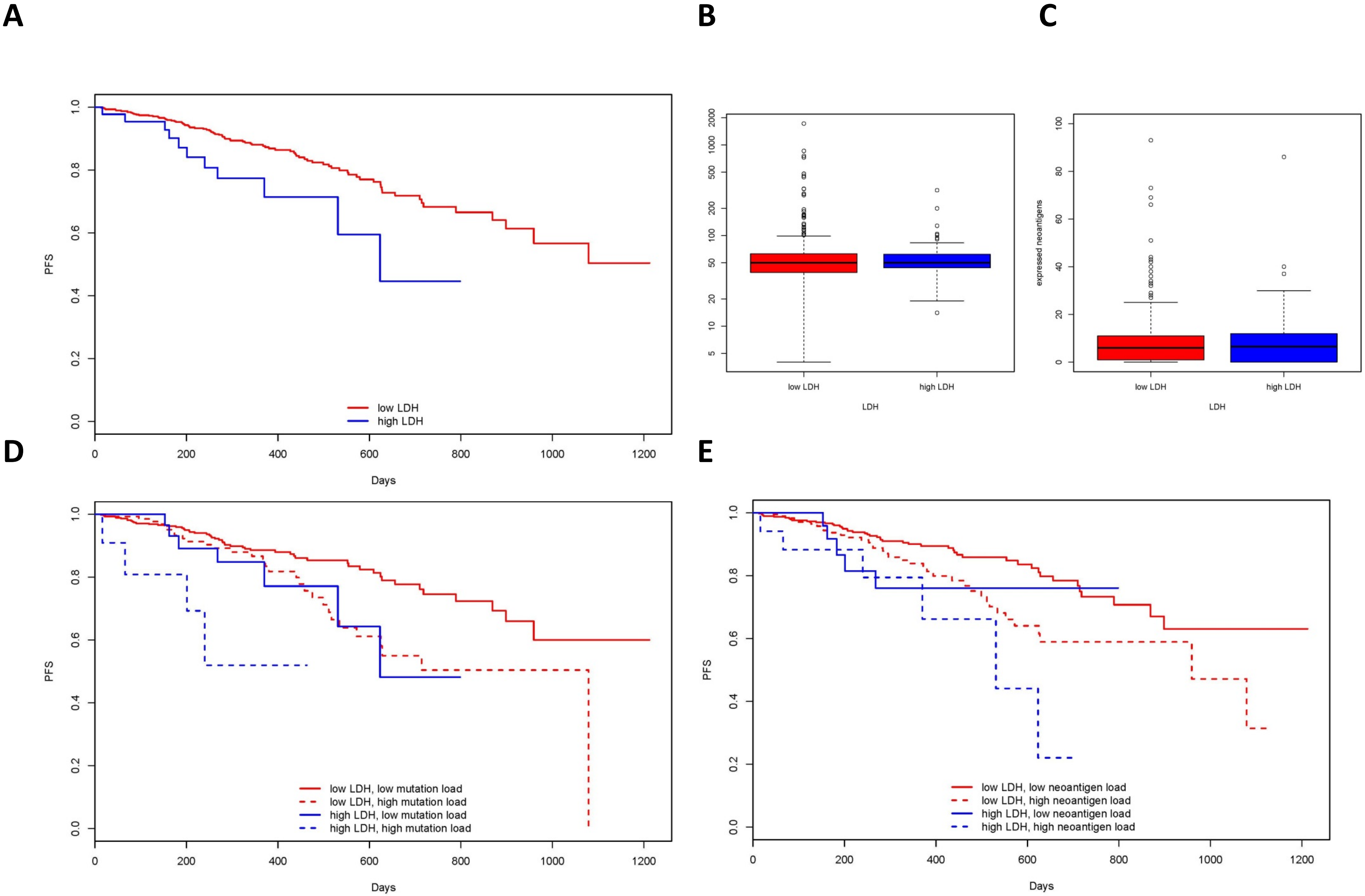

A
B
C
D
E
PFS
Days
low LDH
high LDH
LDH
expressed neoantigens
low LDH, low mutation load
low LDH, high mutation load
high LDH, low mutation load
high LDH, high mutation load
low LDH, low neoantigen load
low LDH, high neoantigen load
high LDH, low neoantigen load
high LDH, high neoantigen load

Figure S6

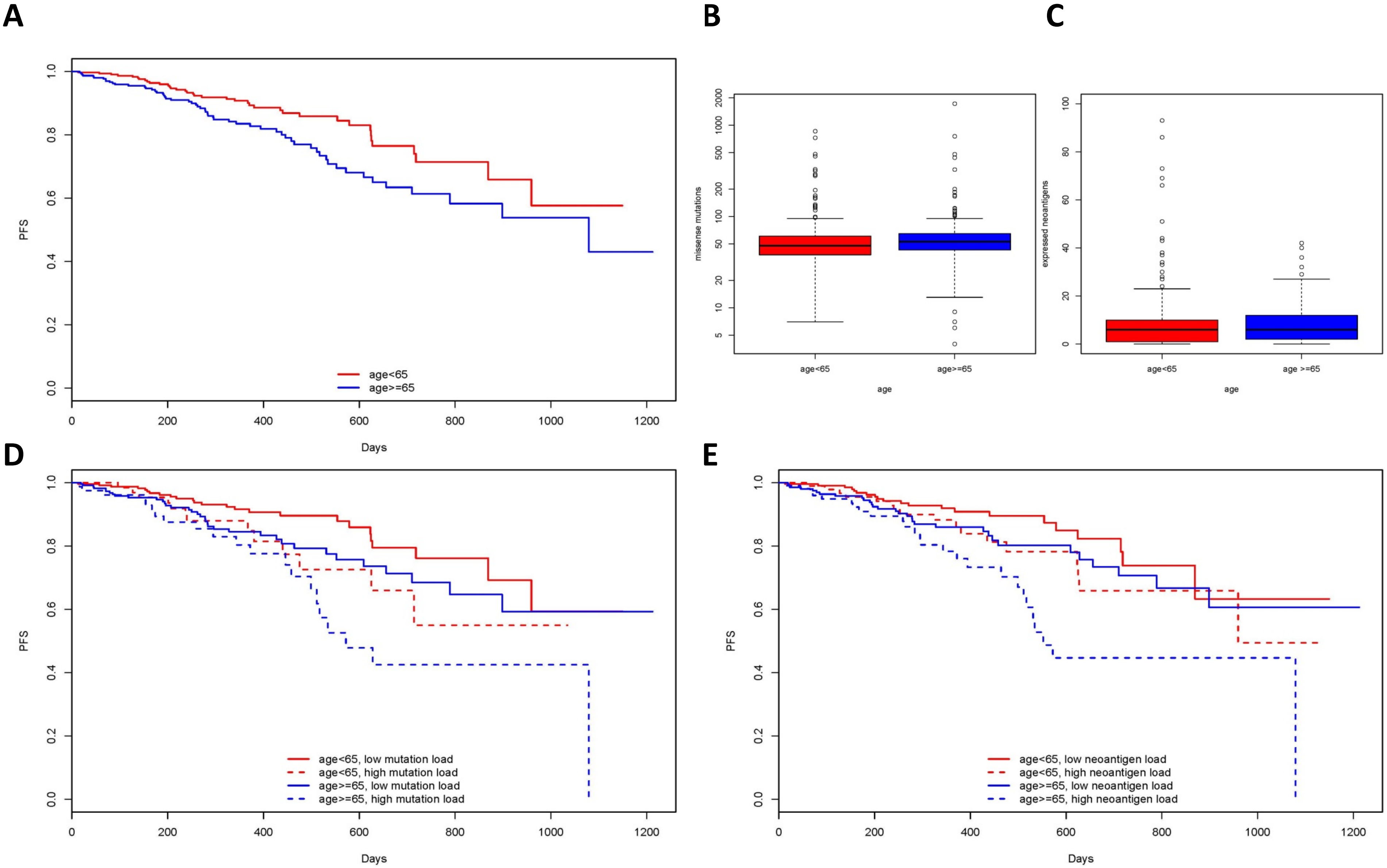

A
PFS
Days
age<65
age>=65
B
missense mutations
age
age<65
age>=65
C
expressed neoantigens
age
age<65
age >=65
D
PFS
Days
age<65, low mutation load
age<65, high mutation load
age>=65, low mutation load
age>=65, high mutation load
E
PFS
Days
age<65, low neoantigen load
age<65, high neoantigen load
age>=65, low neoantigen load
age>=65, high neoantigen load

Table S1

Threshold	2 year survival rate		p-value
**Missense Mutations**	**low**	**high**	
Missense mutations	0.726	0.493	0.00228
missese mutations excluding IG	0.723	0.494	0.00279
**Neoantigens**			
neoantigen	0.709	0.557	0.0637
Expressed neoantigen	0.729	0.555	0.00297
neoantigen excluding IG	0.733	0.481	0.00381
Expressed neoantigens excluding IG	0.691	0.618	0.0737
**Missense mutations + Neoantigens**			
Missense + expressed Nag	0.703	0.488	0.000505
Missense + Nag excluding IG	0.715	0.436	0.00317

Table S2

Threshold	2 year survival rate						p-value
**Disease Stage**	**Stage I**		**Stage II**		**Stage III**		
Stage	0.783		0.669		0.544		1.28E-05
	**Stage I/ low**	**Stage I/high**	**Stage II/low**	**Stage II/high**	**Stage III/low**	**Stage III/high**	
Stage+Missense	0.883	0.452	0.728	0.535	0.559	0.517	6.61E-06
	**Stage I/ low**	**Stage I/high**	**Stage II/low**	**Stage II/high**	**Stage III/low**	**Stage III/high**	
Stage+expressed Nag	0.855	0.661	0.709	0.604	0.64	0.403	2.56e-.06

Table S3

Threshold	2 year survival rate				p-value
**Cytogenetic Abnormalities**	**low risk**		**high risk**		
risk	0.690		0.619		0.522
	**low risk/low**	**low risk/high**	**high risk/low**	**high risk/high**	
risk + missense mutations	0.755	0.315	0.634	0.598	0.00528
	**low risk/low**	**low risk/high**	**high risk/low**	**high risk/high**	
risk + expressed neoantigens	0.731	0.583	0.723	0.532	0.0301

Table S4

<table>
<tr><th>Mutational Load Variable</th><th>Prognostic Risk Factor</th><th colspan="2">Mutational Load Mean (with risk factor)</th><th colspan="2">Mutational Load Mean (without risk factor)</th><th>Significant difference p-value</th></tr>
<tr><td>Missense Mutations</td><td>Cytogenetic high risk</td><td colspan="2">82.47024</td><td colspan="2">57.60887</td><td>0.00539</td></tr>
<tr><td></td><td>1q21amplificaitron</td><td colspan="2">69.20968</td><td colspan="2">60.73317</td><td>0.2121</td></tr>
<tr><td></td><td>Elevated LDH</td><td colspan="2">62.76000</td><td colspan="2">63.99186</td><td>0.8732</td></tr>
<tr><td></td><td>High Age</td><td colspan="2">66.10123</td><td colspan="2">61.77515</td><td>0.5515</td></tr>
<tr><td>Expressed Neoantigens</td><td>Cytogenetic high risk</td><td colspan="2">13.178571</td><td colspan="2">8.114919</td><td>0.01777</td></tr>
<tr><td></td><td>1q21amplificaitron</td><td colspan="2">10.53226</td><td colspan="2">8.71875</td><td>0.3202</td></tr>
<tr><td></td><td>Elevated LDH</td><td colspan="2">9.780000</td><td colspan="2">9.364821</td><td>0.8608</td></tr>
<tr><td></td><td>High Age</td><td colspan="2">9.969325</td><td colspan="2">8.843195</td><td>0.5954</td></tr>
<tr><td></td><td></td><th>Mutational Load Mean (stage I)</th><th colspan="2">Mutational Load Mean (stage II)</th><th>Mutational Load Mean (stage III)</th><td></td></tr>
<tr><td>Missense Mutations</td><td>ISS stage</td><td>67.60563</td><td colspan="2">60.48899</td><td>64.69192</td><td>0.743</td></tr>
<tr><td>Expressed Neoantigens</td><td>ISS stage</td><td>10.88732</td><td colspan="2">8.140969</td><td>9.292929</td><td>0.544</td></tr>
</table>

Table S5

Threshold	2 year survival rate				p-value
**Chromosome 1 Status**	**low risk (no amplification)**		**high risk (1q21 amplification)**		
1q amplification	0.743		0.559		0.000702
	**low risk/low**	**low risk/high**	**high risk/low**	**high risk/high**	
1q amplification + missense mutations	0.752	0.690	0.677	0.377	9.86e-05
	**low risk/low**	**low risk/high**	**high risk/low**	**high risk/high**	
1q amplification + expressed neoantigens	0.762	0.684	0.664	0.424	0.000321

Table S6

Threshold	2 year survival rate				p-value
**LDH**	**low risk (LDH<300IU/L)**		**high risk (LDH>300IU/L)**		
Serum LDH level	0.683		0.446		0.0105
	**low risk/low**	**low risk/high**	**high risk/low**	**high risk/high**	
LDH + missense mutations	0.745	0.504	0.482	0.5191	7.92e-05
	**low risk/low**	**low risk/high**	**high risk/low**	**high risk/high**	
LDH + expressed neoantigens	0.733	0.589	0.760	0.221	0.00151

Table S7

Threshold	2 year survival rate				p-value
**Age**	**low risk (age<65)**		**high risk (age ≥65)**		
Age	0.714		0.613		0.0115
	**low risk/low**	**low risk/high**	**high risk/low**	**high risk/high**	
Age + missense mutations	0.761	0.549	0.685	0.425	0.00129
	**low risk/low**	**low risk/high**	**high risk/low**	**high risk/high**	
Age + expressed neoantigens	0.738	0.659	0.707	0.447	0.000435

Table S8

Univariate	HR	p–value
**Chromosome 1 Status**		
1q21 amplification	2.0	<0.01
**LDH**		
High LDH	2.2	0.01
**Age**		
Age>=65	1.7	0.01
**Multivariate**		
**Chromosome 1 Status + Missense Mutations**		
1q21 amplification	1.8	0.01
+ missense mutations	1.7	0.02
**Chromosome 1 Status + Neoantigens**		
1q21 amplification	1.8	<0.01
+ expressed neoantigens	1.7	0.01
**LDH + Missense Mutations**		
High LDH	2.4	0.01
+ missense mutations	2.0	<0.01
**LDH + Neoantigens**		
High LDH	2.1	0.02
+ expressed neoantigens	1.8	<0.01
**Age + Missense Mutations**		
Age>=65	1.7	0.02
+ missense mutations	1.9	<0.01
**Age + Neoantigens**		
Age>=65	1.7	0.01
+ expressed neoantigens	1.9	<0.01

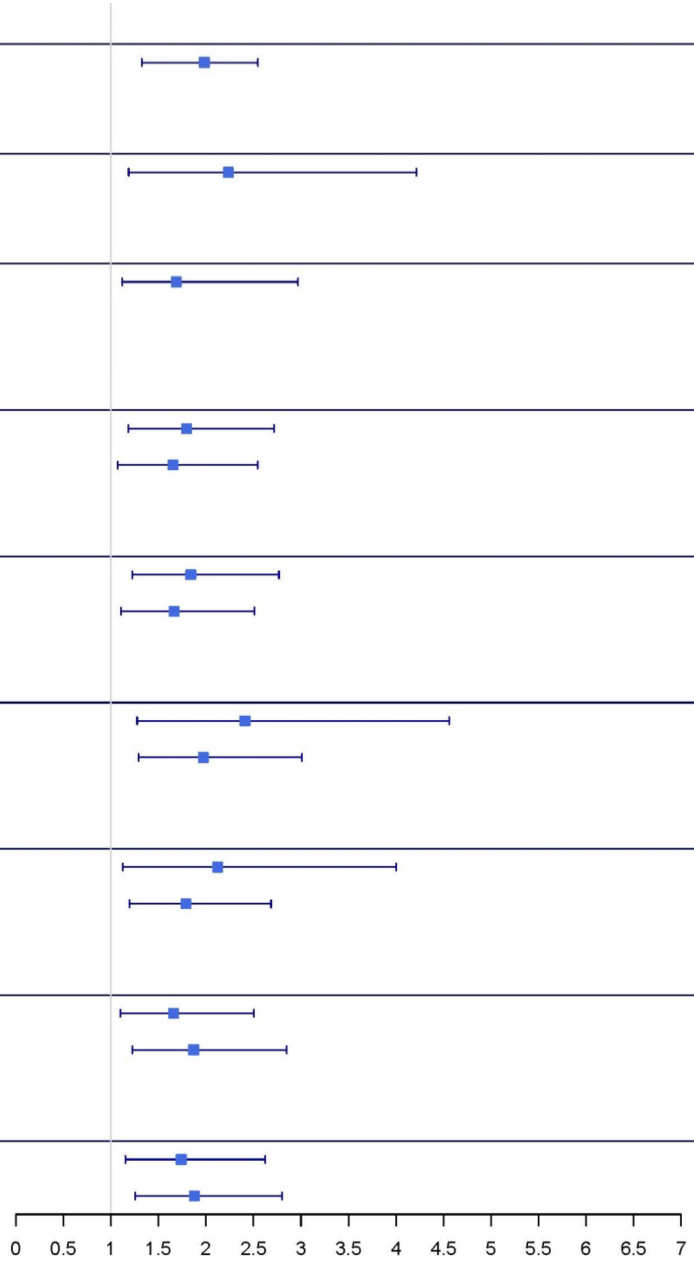

Supplement: Supplementary Figures [file bcj201794x2.pdf]
